# Comparative Analysis of *piggyBac,* CRISPR/Cas9 and TALEN Mediated BAC Transgenesis in the Zygote for the Generation of Humanized *SIRPA* Rats

**DOI:** 10.1038/srep31455

**Published:** 2016-08-17

**Authors:** Chris J. Jung, Séverine Ménoret, Lucas Brusselle, Laurent Tesson, Claire Usal, Vanessa Chenouard, Séverine Remy, Laure-Hélène Ouisse, Nicolas Poirier, Bernard Vanhove, Pieter J. de Jong, Ignacio Anegon

**Affiliations:** 1 Center for Genetics, Children’s Hospital Oakland Research Institute, CA 94609, Oakland, USA; 2 Platform Rat Transgenesis Immunophenomic, SFR Francois Bonamy, CNRS UMS3556 Nantes, F44093, France; 3 INSERM UMR 1064-ITUN; CHU de Nantes, Nantes F44093, France; 4 OSE Immunotherapeutics, 44000 Nantes, France

## Abstract

BAC transgenic mammalian systems offer an important platform for recapitulating human gene expression and disease modeling. While the larger body mass, and greater genetic and physiologic similarity to humans render rats well suited for reproducing human immune diseases and evaluating therapeutic strategies, difficulties of generating BAC transgenic rats have hindered progress. Thus, an efficient method for BAC transgenesis in rats would be valuable. Immunodeficient mice carrying a human *SIRPA* transgene have previously been shown to support improved human cell hematopoiesis. Here, we have generated for the first time, human *SIRPA* BAC transgenic rats, for which the gene is faithfully expressed, functionally active, and germline transmissible. To do this, human *SIRPA* BAC was modified with elements to work in coordination with genome engineering technologies-*piggyBac*, CRISPR/Cas9 or TALEN. Our findings show that *piggyBac* transposition is a more efficient approach than the classical BAC transgenesis, resulting in complete BAC integration with predictable end sequences, thereby permitting precise assessment of the integration site. Neither CRISPR/Cas9 nor TALEN increased BAC transgenesis. Therefore, an efficient generation of human *SIRPA* transgenic rats using *piggyBac* opens opportunities for expansion of humanized transgenic rat models in the future to advance biomedical research and therapeutic applications.

Mammalian model systems provide an essential platform in biomedical research for deciphering the complexities underlying the pathogenesis of human disease, and for developing the applicative and translational potential of new therapies. Bacterial artificial chromosomes (BACs) have played an important role in these endeavors by providing the DNA source material with which transgenic animals are derived. BACs are *E. coli* based large insert DNA clones capable of carrying genomic fragments ranging in size between 150–300 kb^1^,^2^. Unlike transgenic animals created using small plasmids, the large insert size of BACs allows for the transgene to maintain stability and embody low chimerism^3^. Moreover, the inserts typically include enhancer and other regulatory elements, minimizing the undesirable consequences of position-effects, such as epigenetic silencing and unexpected splicing^2^,^4^,^5^. For these reasons, the past decade has witnessed a rapid growth of transgenic mice generated using BACs, rendering it the preferred method for creating animal models recapitulating human gene expression and disease modeling.

While the large genomic insert size of BACs is beneficial for creating animals with transgenes that are integration site independent and accurately expressed contingent on copy number *in vivo*, this quality also makes efficient integration of BACs into host genomes challenging. Furthermore, the BAC integration site is difficult to determine. In spite of these limitations, BACs continue to be widely used, mainly for the production of transgenic mice. In contrast, BAC transgenesis in rats has been less successful, due to inherent physiological differences between the two rodent species^6^, leading to barriers at the level of rat oocyte manipulation and ES cell derivation and expansion^7^. These obstacles have been unfortunate, as they hinder progress towards the applicative potential of the rat model system, which harbor distinct advantages over the mouse. For example, the rat offers the short generation time and cost effectiveness of the mouse model, yet, in contrast to their smaller counterparts, the rat is genetically and physiologically more similar to the human^8^, and the larger body mass is poised to yield more biological material per animal unit that can be harvested in the course of experimentation.

An area of research where BAC transgenic rat model system would be particularly useful is the humanization of the immune system. While immune-deficient mouse and rat strains devoid of key lymphoid components have played an important role in biomedical sciences by making feasible studies involving the engraftment of human cells, tissues and organs^9^,^10^,^11^,^12^, empirical evidence has shown that the absence of lymphoid cells alone is not sufficient for efficiently modulating the engraftment of human cells^13^. In 2007, Takenaka and colleagues^14^ reported for the first time that in the lymphocyte depleted non-obese diabetic (NOD) severe combined immunodefiency (SCID) mouse strain^15^, NOD strain specific polymorphisms in the signal-regulatory protein alpha (*Sirpa*) locus supported human hematopoiesis through enhanced binding of the SIRPα protein to the human CD47 ligand. Once bound, SIRPα acts as an inhibitory receptor for mouse macrophages, allowing human hematopoietic cells to escape host cell phagocytosis^16^,^17^,^18^,^19^. To confirm that the enhanced engraftment of human cells in NOD strains is dependent on polymorphic SIRPα, thereby ruling out the possibility of causation or influence by strain specific extraneous factors, Strowig *et al*.^20^ generated human *SIRPA* BAC transgenic mice on mixed 129/BALB/c background, and demonstrated that the expression of human *SIRPA* enhanced human cell engraftment and improved functionality of human adaptive immune system *in vivo*. For these reasons, an efficient method for generating human *SIRPA* BAC transgenic rats would allow for the building of a repository of humanized *SIRPA* rats on various immune-deficient rat strains^21^, for use as a tool for studying the engraftment potential of human cells and tissues, as well as for reproducing human immune diseases and evaluating therapeutic strategies.

Here, we aim to generate BAC transgenic rats faithfully expressing human SIRPα. To do this, we seek to develop strategies utilizing genome engineering technologies reported to be highly efficient for generating transgenic animal models. Specifically, we examined *piggyBac* transposon, CRISPR/Cas9 and TALEN mediated approaches, as they have emerged as powerful tools for manipulating the genome. The *piggyBac* transposon system is a genetic element capable of mobilizing a segment of DNA encased between terminal inverse repeat (TIR) elements in the presence of transposase proteins^22^,^23^,^24^. The mobilized DNA is then transpositioned into a TTAA site in a different location in the genome by the transposase, for which the insertion location can be precisely determined using PCR^25^,^26^,^27^. Taking advantage of the *piggyBac* system’s “cut and paste” mechanism, researchers have utilized the TIR elements to design strategies for carrying out high throughput insertional mutagenesis for cancer research^28^,^29^, cellular reprogramming of stem cells^30^,^31^, among a slew of other experimentations requiring genome engineering^32^,^33^. Of relevance to this study is a recent pioneer publication showing that BACs retrofitted with TIR elements can be efficiently transposed in mouse zygotes^34^.

In contrast to the *piggyBac* mediated approach, CRISPR/Cas9 and TALEN are a family of endonucleases capable of inducing double stranded break (DSB) at precise locations in the genome, initiating the stimulation of two different pathways of repair mechanism–non-homologous end-joining (NHEJ) and homology-directed repair (HDR)^35^,^36^,^37^,^38^,^39^,^40^. The ability to stimulate NHEJ and HDR by CRISPR/Cas9 and TALEN has ushered in an era in which precise editing of the genome with high efficiency has become possible. However, CRISPR/Cas9 has very recently been described in a manuscript, submitted concomitantly with our study, to support targeted integration of a single BAC^41^. TALENs have not yet been reported for targeted BAC integration in animals. In advancement of this field, we showed^42^ recently that in both mouse and rat models, CRISPR/Cas9 and TALEN were successfully used for targeting an eGFP expressing vector, approximately 4.5 kb in size, into the *Rosa26* locus through HDR.

Encouraged by these findings, we seek to modify the human *SIRPA* carrying BAC to work in coordination with *piggyBac* transposition, CRISPR/Cas9 or TALEN mediated approaches for the generation of humanized BAC transgenic rats. Hence, the objective of this study is twofold: 1) to develop an efficient method for generating BAC transgenic rats by evaluating the *piggyBac*, CRISPR/Cas9 and TALEN mediated approaches in the zygote; and 2) to generate humanized *SIRPA* rats that are functionally viable by expressing the protein in leukocytes and actively interacting with human CD47 ligand.

## Results

### Conversion of a human *SIRPA* carrying BAC into a *piggyBac* transposon

**Figure 1:**
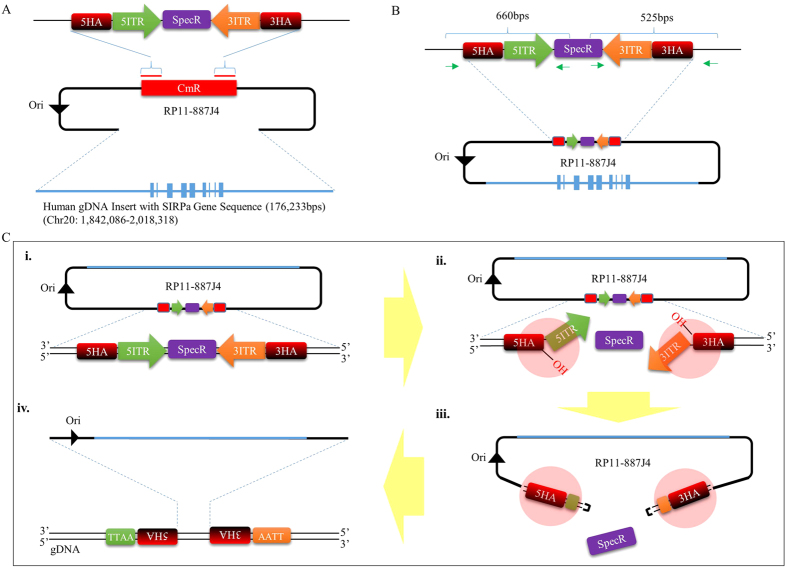
Strategy for converting *hSIRPA*-BAC DNA into a *piggyBac* transposon. (**A**) Diagram illustrating the strategy used for retrofitting hSIRPA-BAC DNA (RP11-887J4) with *piggyBac* TIR elements. 5′ TIR (green) and 3′ TIR (orange) elements were sub-cloned into pUC19 vector backbone with spectinomycin resistance gene (purple), and 50 bp homology arm sequences (red) used for replacing the chloramphenicol resistance gene in the BAC vector backbone via recombineering technology. The diagram also indicates that the genomic DNA insert in the RP11-887J4 BAC is 176,233 bps, covering the SIRPA genic region, on chromosome 20 between 1,842,086-2,018,318. (**B**) The green arrows indicate the primer pairs used to verify hSIRPA-BAC retrofitting after the recombineering process. (**C**) A schematic diagram describing the transpositioning strategy of hSIRPA-BAC retrofitted with TIR elements mediated by *piggyBac* transposase. Illustration (i) shows the retrofitted BAC DNA. Illustrations (ii) and (iii) show the process by which the *piggyBac* transposase proteins bind to the TIR sequences, initiating nicking of the DNA strands, allowing 3′ hydroxyl group at both ends of the transposon to hydrophilic attack the flanking TTAA sequence and freeing the BAC from the spectinomycin resistance gene by forming hairpin structure at the TIR ends. Once the BAC DNA is released from spectinomycin resistance gene, illustration (iv) shows repairing of the linearized BAC DNA by ligating into the complementary TTAA overhangs in the genomic DNA through the mediation of the *piggyBac* transposase proteins.

The initial steps required for developing a *piggyBac* mediated BAC transposition system involves converting the large circular DNA into a transposon capable of being recognized and mobilized by the transposase proteins (Supp. Fig. 1). To do this, we selected a BAC clone (RP11-887J4) (Supp. Fig. 2) containing 176 kb of human genomic DNA segment covering the *SIRPA* transcript coding sequence, as well as the region spanning 52 kb bps up- and 78 kb down-stream of the transcript start and end points. The selected human *SIRPA* containing BAC was then retrofitted with a cassette containing the 5′ and 3′ *piggyBac* Terminal Inverted Repeat (TIR) elements flanking the spectinomycin resistance gene, by replacing the chloramphenicol resistance gene in the vector backbone (Fig. 1A) (from this point onward, the TIR retrofitted *hSIRPA*-BAC will be referred to as *hSIRPA-BAC-TIRs DNA*). The positioning of the TIR elements in the BAC vector backbone was designed to mimic the inherent ‘cut and paste’ mechanism of *piggyBac*-based gene transfer, for which DNA segment encapsulated between the two TIR elements is seamlessly excised out of the genome in the presence of the transposase proteins, and transpositioned into a different location in genomic sites containing the TTAA sequence (Supp. Fig. 1). In the case of the *hSIRPA*-BAC-TIRs DNA, the TIR sequences are oriented such that in the presence of the transposase proteins, the 5′ and 3′ TIR elements would be excised away from the spectinomycin resistance gene, allowing the mobilization of the entire BAC for transposition into one of the TTAA sequences in the rat genome (Fig. 1C). In this strategy, when the mRNA transcript of the *piggyBac* transposase is co-microinjected into the rat zygote with the *hSIRPA*-BAC-TIRs DNA, the transcribed transposase proteins bind to the inverted repeats in order to induce nicks at both ends of the transposon, exposing the 3′ hydroxyl group and TTAA tetranucleotide overhang in the opposite strands (Fig. 1C). The 3′ hydroxyl end launches a hydrophilic attack on the TTAA overhang, creating a hairpin formation, which initiates the release of the *hSIRPA*-BAC-TIRs DNA from the spectinomycin resistance gene. Once released, the hairpin structure is resolved by leaving TTAA overhang at the 5′ ends of the transposon, and 3′ hydroxyl group exposed at the opposite strands. During this process, the transposase proteins identify other TTAA sequences in the genome, and likewise, induce nicks and hydrophilic 3′ hydroxyl attacks on the TTAA tetranucleotide sequences. The resolution of the hairpin formation in the genome involves target joining of the 3′ hydroxyl groups at the transposon ends, with the 5′ staggered TTAA overhanging sequence in the genomic DNA. Through this process, the *hSIRPA*-BAC-TIRs DNA transposon gets inserted into the genome, by duplicating the TTAA sequence, and positioning itself such that each tetranucleotide sequence ends up residing at opposite ends of the transpositioned *hSIRPA*-BAC-TIRs.

### Generation of *piggyBac* mediated humanized *SIRPA* rats via zygote injection

**Figure 2:**
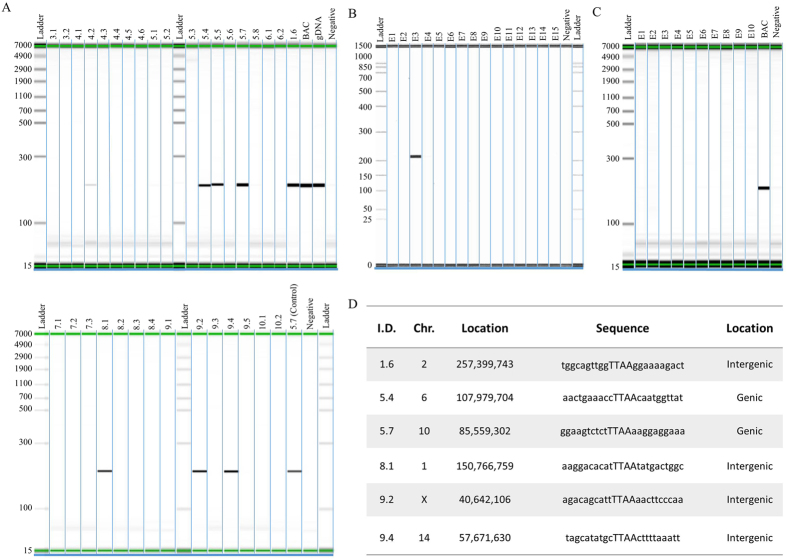
Analysis of *piggyBac* mediated *hSIRPA*-BAC-TIR transposition. (**A**) Genotyping result showing PCR positive pups for a 201 bp regions in the human SIRPA gene. The diagram shows pup numbers 1.6, 4.2, 5.4, 5.5, 5.6, 5.7, 8.1, 9.2 and 9.4 to be positive for PCR. In the upper panel, BAC refers to RP11-887J4 used as a positive control, gDNA refers to human genomic DNA as a positive control, and Negative as a negative control. In the lower panel, 5.7 (Control) refers to genomic DNA from pup number 5.7 used as a positive control. (**B** and **C**) Show PCR result of rat zygotes injected with Cas9 mRNA transcript, instead of *piggyBac* transposase, as a negative control. BAC refers to RP11-887J4 used as a positive control. To determine transposition, splinkerette PCR was performed in order to sequence the region adjacent to the TIR ends. (**D**) Shows the sequencing result, indicating that six pups (1.6, 5.4, 5.7, 8.1, 9.2 and 9.4) carry transpositioned hSIRPA-BACs. The first column shows the pup identities, the second and third columns show the location of the transposition sites, the fourth column lists the TTAA site of transposition, and sequences ten base up and downstream. The last column indicates whether the transposition occurred in genic or intergenic region.

The implementation of the *piggyBac* mediated *hSIRPA*-BAC-TIRs DNA transposition strategy was carried out by co-injecting 2 or 3 ng/ul of BAC DNA with 25, 50 or 100 ng/ul of hyperactive transposase (hyPBase) mRNA into the pronucleus of rat zygotes (Table 1). Co-injections of 25 ng/ul of hyPBase and 2 ng/ul of BAC yielded 25 pups (13.8% relative to the number of embryos transferred), 50 ng/ul of hyPBase and 3 ng/ul of BAC yielded 7 pups (13.1% relative to the number of embryos transferred), and 100 ng/ul of hyPBase and 3 ng/ul of BAC yielded 13 pups (36.8% relative to the number of embryos transferred). To evaluate the efficiency of BAC insertion in these pups, tissue biopsies were performed on the tails for genomic DNA isolation, which was used to genotype for the presence of the human *SIRPA* gene (Supp. Table 1). In both conditions with either 50 ng/ul or 100 ng/ul of hyPBase, 1 pup was found to be PCR positive (14.3% and 7.7%, respectively, relative to the total number of pups), while 6 pups were found to be PCR positive in the 25 ng/ul of hyPBase condition (24.0% relative to the total number of pups) (Table 1 and Fig. 2A). As a control experiment, we replaced the hyPBase mRNA for the Cas9 mRNA. Cas9 in the absence of sgRNA will not cleave the DNA^39^ and thus would essentially be a bystander mRNA to be co-injected with the *hSIRPA*-BAC-TIRs. Because of the higher percentage of PCR positive pups relative to the total number of pups in the condition in which 2 ng/ul of BAC and 25 ng/ul of hyPBase mRNA transcripts were used, negative controls were set up using 2 ng/ul of *hSIRPA*-BAC-TIRs DNA and 25 ng/ul of Cas9 mRNA transcripts (Fig. 2C,D and Table 1). In the negative control experiment, 25 embryos (E15) were genotyped (32.1% relative to the number of embryos transferred), of which 1 embryo was found to be PCR positive (1.3% relative to the total number of embryos transferred) (Table 1). These findings suggest that: 1) co-injecting *hSIRPA*-BAC-TIRs DNA with hyPBase *piggyBac* increases the number of pups that are PCR positive for the presence of *hSIRPA*; and 2) while increasing the amount of hyPBase co-injected with 100 ng may give rise to higher number of pups relative to the number of embryos injected, the genotyping result indicates a higher percentage of PCR positive pups relative to the total number of pups when the amount of hyPBase injected is decreased to 25 ng.

### Identification of transposition sites in the genome

Having shown that co-injecting the *piggyBac* TIR retrofitted *hSIRPA* BAC with hyPBase mRNA transcripts increases the number BAC insertions relative to the negative control, we sought to determine whether these insertions are transposition events mediated by *piggyBac* transposase. To do this, splinkerette PCR was utilized to: 1) identify the precise location of the insertion sites; and 2) determine whether the BAC transposon ends are flanked by TTAA sequence, since the hallmark feature of *piggyBac* mediated transposition is the mobilization of the transposon from one TTAA region into another, by duplicating the tetranucleotide sequence, and inserting itself in between the duplicated sequences (Supp. Figs 1 and 3). Among the 8 pups genotyped to be positive for *hSIRPA* BAC insertions (1.6, 4.2, 5.4, 5.5, 5.7, 8.1, 9.2, 9.4), 6 were found to have duplicated TTAA sequences, each tetranucleotide residing immediately adjacent to the 5′ and 3′ TIR elements (1.6, 5.4, 5.7, 8.1, 9.2, 9.4) (Fig. 2D). Detailed analysis of the integration sites show that pups 1.6, 8.1, 9.2 and 9.4 have *hSIRPA*-BACs transpositioned at intergenic regions, whereas, pups 5.4 and 5.7 have BAC transpositioned at genic regions, *Ppp2r5e* and *Lasp1*, respectively (Fig. 2D and Supp. Fig. 4a–f).

### Validation of transpositioned BAC integrity

**Figure 3:**
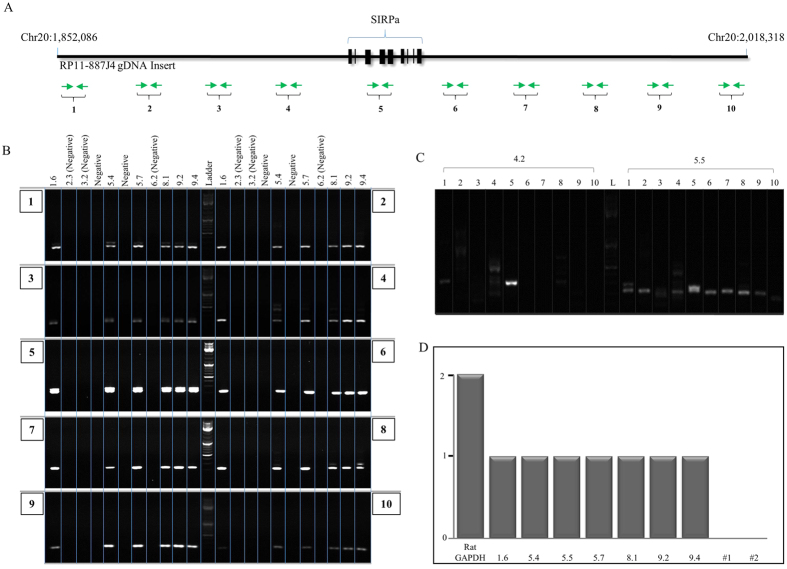
Integrity of transpositioned *hSIRPA*-BACs and copy number analysis. (**A**) Graphic illustration showing the genomic DNA insert in RP11-887J4. The chromosomal locations of start and end points are indicated. Exon in the SIRPA gene are blocked in bold. Green arrows indicate the primer pairs (10) spanning along entire BAC at approximately 20 kb intervals, used to verify the presence of the full BAC in pups confirmed to have hSIRPA-BAC transpositioned. (**B**) PCR result showing that six pups (1.6, 5.4, 5.7, 8.1, 9.2 and 9.4) with transpositioned hSIRPA-BACs are confirmed to be positive for all ten primer pairs. Genomic DNAs from pups 2.3, 3.2 and 6.2 were used as negative controls (Fig. 2A). (**C**) PCR result two pups (4.2 and 5.5) that were found to be positive for the presence of the human SIRPA gene during the genotyping screening, but negative for *piggyBac* mediated transposition. The results show that pup 4.2 is positive only for primer pair 5, suggesting random insertion of a fragmented BAC DNA. Pup 5.5 is found to be positive for all ten primer pairs, suggesting random insertion of hSIRPA-BAC DNA. (**D**) Bar graph showing RT-qPCR result indicating the copy number of human SIRPA BAC in *piggyBac* transpositioned founders (1.6, 5.4, 5.7, 8.1, 9.2, 9.4), random insertion founder (5.5), two negative controls (#1, #2). RT-qPCR results were analyzed relative to the rat GAPDH, for which there are two copies in the genome.

To determine whether the transpositioned *hSIRPA*-BAC-TIRs DNA are fully intact, a series of primer pairs were designed at approximately 20 kb intervals, along the BAC insert (Fig. 3A, Supp. Table 1). Polymerase chain reactions showed that all the transpositioned BACs in pups 1.6, 5.4, 5.7, 8.1, 9.2, 9.4 are fully intact (Fig. 3B). Analysis of the two pups (4.2 and 5.5) that were genotyped to be PCR positive for a section of the human *SIRPA* gene, but negative for transposition, showed that: 1) pup 4.2 was PCR positive only at primer pair 5, suggesting random integration of a fragmented BAC consistent with the absence of TIRs; whereas 2) pup 5.5 showed random insertion of a full BAC DNA without transposition (Fig. 3C). However, the results for pup 5.5 do not establish how the circular BAC was disrupted during the random integration, and it remains to be established if the *hSIRPA* locus is present as one contiguous segment. Our findings indicate that converting the circular BAC DNA into a large *piggyBac* transposon is an efficient and robust approach for generating transgenic rats, for which the transposition site can be precisely determined, and the likelihood of a complete insert is increased.

### Analysis of copy number integration

Unlike transgenic rodents generated using small plasmids where multiple copies of transgene can be integrated leading to abnormally high expression of the transgene, BAC transgenics tend to have a single copy integration, thereby more accurately reflecting normal physiological level of transgene expression. To evaluate whether *piggyBac* mediation effects copy number integration of BAC DNA, we further characterized the transposition positive founders (1.6, 5.4, 5.7, 8.1, 9.2, 9.4), as well as the pup with random *hSIRPA*-BAC-TIRs DNA insertion (5.5), using qPCR on genomic DNA. When normalized to the C_t_ values derived from the endogenous rat *Gapdh* gene, for which there are two copies, we found that all of the founders carry a single copy of BAC integration (Fig. 3D).

### Analysis of germline transmission

Having shown that the transgenic founder pups generated by *piggyBac* transposition events carry a single copy of the full length *hSIRPA*-BAC-TIRs DNA, we sought to determine the germline transmission efficiency. To do this, *hSIRPA*-BAC-TIRs DNA founders (with the exception of 8.1, which died prior to mating) were mated with wild-type animals to obtain F1 offspring, and analyzed for the rate of BAC transmission (Table 2). PCR genotyping of offspring revealed that founders 1.6 and 9.4 transmitted the BAC to the F1 offspring in a Mendelian manner, whereas founder 5.7 transmitted in a low proportion of the offspring. Founder 4.2 with a fragmented BAC insertion transmitted to the offspring as well. Founders 5.4, 5.5 and 9.2 on the other hand, did not transmit the transgene to their offspring. These analyses indicate that 3 out of 5 of viable *hSIRPA*-BAC-TIRs DNA transgenic founders derived from *piggyBac* transposition events transmitted the BAC transgene to their F1 offspring, whereas 2 out of 5 may likely be mosaics resulting in failure of transmission.

### Functional analysis of *hSIRPA* expression in BAC transgenic rats

**Figure 4:**
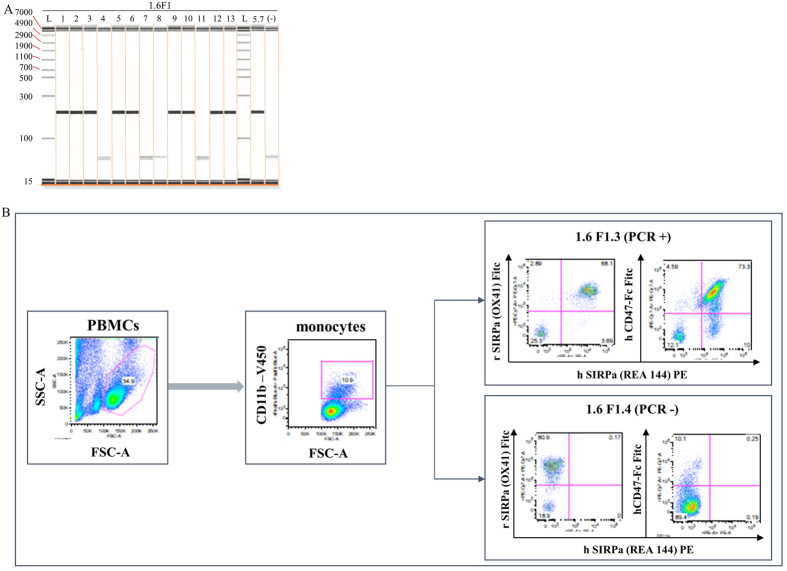
Analysis of 1.6 F1 offspring for BAC transmission and *hSIRPA* protein expression. (**A**) The PCR analysis shows human *SIRPA* BAC detection in 9 out of 13 offspring from the founder 1.6. Genomic DNA from founder 5.7 was used as a positive control. (**B**) Animals 1.6 F1.3 and 1.6 F1.4 are F1 offspring animals representative of PCR *SIRPA*-BAC positive or negative animals, respectively. Peripheral blood mononuclear cells (PBMCs) were first gated by morphology (dot plot SSC-FSC), then CD11b+ cells (mostly expressed by monocytes) were labelled with a rat anti-CD11b antibody (dot plot CD11b-FSC) and finally, cells were stained with combinations of human and rat anti-SIRPα antibodies (left dot-plots) or human anti-SIRPA antibodies and human CD47-Fc (right dot-plots). The large majority of rat monocytes from BAC-*SIRPA*-TIR^+^ transgenic rats expressed human SIRPα, as detected using both anti-human SIRPα antibodies and human CD47-Fc whereas all monocytes from BAC-*SIRPA*-TIR^−^ transgenic rats were negative for both labels.

In order to evaluate the functionality of *hSIRPA* in the transpositioned BAC DNA, thirteen F1 offspring derived from founder 1.6 were analyzed first for the presence of the transgene (Fig. 4A) and then for the expression of human SIRPα on CD11b+ monocytes, using a human specific CD47-Fc fusion molecule, which binds to human, but not rat Sirpα. All F1 animals positive for the transgene bound human CD47-Fc, but none did among F1 animals negative for the transgene, as shown for two representative animals from each group (Fig. 4B and data not shown). The rationale for evaluating CD11b+ cells among rat leukocyte populations stems from the fact that among human^17^ and rat^43^ peripheral blood mononuclear cells, SIRPα expression is mainly restricted to monocytes (essentially all CD11b^+^) (data not shown). Furthermore, in mice transgenic for human SIRPα expression, human SIRPα detection was restricted to mouse monocytes^20^, and mononuclear phagocytes are the main cells involved in clearing human cells in immune humanized mice^17^.

We also evaluated the expression of hSIRPα in rat monocytes by staining with anti-human and rat specific SIRPα monoclonal antibodies. The results confirmed that genotyped positive offspring (1.6F1.3) faithfully expressed both human and rat SIRPα protein on CD11b^+^ cells, while the genotyped negative offspring (1.6F1.4) showed expression of only the rat SIRPα protein (Fig. 4B). Additional analysis using SE5A5 monoclonal antibody confirmed our findings (data not shown). Interestingly, all monocytes expressing human SIRPα also expressed rat SIRPα (Fig. 4B), suggesting a common set of underlying molecular mechanisms governing the expression of both human and rat *SIRPA* gene, and supporting the future use of human SIRPα transgenic and deficient for *Rag1*^21^ and *Il2rg* (unpublished) genes as recipients for immune humanization experiments. These findings indicate that *piggyBac* mediated *hSIRPA*-BAC-TIRs transpositioned rats give rise to progeny that faithfully express functionally viable human SIRPα protein in rat monocytes.

### Generation of CRISPR/Cas9 and TALEN mediated hSIRPA-BAC transgenic rats

We have thus far shown the efficiency with which functionally viable humanized *SIRPA* transgenic rats can be generated when mediated by *piggyBac* transposition. While the BAC insertion sites can be readily determined using splinkerette PCR, one of the major limitations of the *piggyBac* mediated approach is that the BAC can be inserted anywhere in the genome with a TTAA tetranucleotide sequence. From this vantage point, both CRISPR/Cas9 and TALEN endonucleases offer the potential benefit of targeting the BAC into a specific location in the genome. To test this possibility, we designed a strategy for applying the same CRISPR/Cas9 and TALENs, that we previously used^42^ for inducing DSB in the rat *Rosa26* locus, to stimulate HDR^44^. To take advantage of the HDR repair mechanism, we modified the *hSIRPA* BAC by retrofitting a cassette containing 900 base pairs of *Rosa26* homology arm with sequences flanking the endonuclease cut site. In our laboratories, we have shown that 900 base pair homology arm length is sufficient for targeting circular KOMP vectors (approximately 9 kb) in the mouse genome at high efficiency (data not shown), and that 4.5 kb linearized constructs with similar homology arms can be targeted using CRISPRs and TALENs to *Rosa26* and *Hprt* in mouse and rat zygotes^42^. Two different cassettes were designed such that one contains the same rat *Rosa26* sgRNA or TALEN recognition sequence separating the 5′ and 3′ homology arms, and the other cassette that lacks the recognition sequence. By designing cassettes with or without the sgRNA and TALEN recognition sequences, the aim was to simultaneously linearize or keep the BAC circular in the injected embryo when the endonuclease cleaved the genomic DNA at the targeted *Rosa26* locus. The *hSIRPA*-BAC that was retrofitted with the linearization site will be referred to as *hSIRPA*-BAC-Rosa26^+^, and the BAC without the linearization site will be referred to as *hSIRPA*-BAC-Rosa26^−^.

The modified BACs were microinjected with the Rosa26 sgRNA and Cas9 protein because we previously demonstrated that Cas9 protein was more efficient than mRNA to obtain genome insertion of donor DNA^42^. Microinjection with the *hSIRPA*-BAC-Rosa26^+^ did not show toxic effects on embryo survival immediately before microinjection (76.3% viable), yielded 60 day-15 fetuses (40.5% relative to the number of zygotes transferred), resulted in 1 founder PCR positive for the BAC (1.7% of fetuses) and 22 founders (36.7% of fetuses) with mutations around the cleavage site in the Rosa26 locus (Table 3). Microinjection with the *hSIRPA*-BAC-Rosa26^−^ yielded roughly similar results, no toxic effects on embryo survival immediately before microinjection (69.6% viable), yielded 28 day-15 fetuses (32.2% relative to the number of zygotes transferred), resulted in 1 founder PCR positive for the BAC (3.6% from fetuses) and in 12 founders (42.8% from the fetuses) with mutations around the cleavage site of the *Rosa26* locus (Table 3). The founder obtained with *hSIRPA*-BAC-Rosa26^+^ did not show insertion in the *Rosa26* locus as depicted by negative junction PCRs (data not shown). Therefore, despite that sgRNAs for the Rosa26 locus were highly active, as evidenced by high rates of mutations, indicating that the *hSIRPA*-BAC-Rosa26^+^ was likely linearized, the frequency of transgenic BAC rats was not increased as compared to the one observed using the circular *hSIRPA*-BAC-Rosa26^−^.

We also tested the microinjection of the *hSIRPA*-BAC-Rosa26 with TALE nuclease mRNA which we previously showed to efficiently and reproducibly target plasmid donor DNA integration by HDR in the *Rosa26* locus^38^. Microinjection with the *hSIRPA*-BAC-Rosa26^+^ did not show toxic effects on embryo survival immediately before microinjection (68.9% viable), yielded 39 day-15 fetuses (32% relative to the number of zygotes transferred), resulted in no founders PCR positive for the BAC (0% of fetuses), and in 9 founders (23.1% of fetuses) with NHEJ mutations around the cleavage site of the *Rosa26* locus (Table 3). Microinjection with the *hSIRPA*-BAC-Rosa26^−^ yielded roughly similar results, no toxic effects on embryo survival immediately before microinjection (66% viable), yielded 13 day-15 fetuses (19.7% relative to the number of zygotes transferred), resulted in 0 founder PCR BAC positive (0% from fetuses) and in 4 founders (30.8% from the fetuses) with mutations around the cleavage site of the *Rosa26* locus (Table 3). Thus, the TALENs also did not result in integration of the BAC in the targeted locus.

## Discussion

In this study, we have generated for the first time: 1) highly efficient *piggyBac* mediated BAC transgenic rats that are germline transmissible; and 2) rats faithfully expressing functionally viable human SIRPα. While the procedures involved in the production of transgenic rats and mice are similar, the efficiency of creating transgenic rats has remained more difficult than mice^6^,^45^. This difficulty is attributed to inherent physiological differences between the two rodent species, giving rise to lower transformation rate of microinjected pronuclear stage zygotes in rats relative to mice. Moreover, unlike in mice, the derivation of germline competent rat embryonic stem (ES) cells could not be achieved until only recently with the discovery of MEK, GSK-3 and FGFR inhibiting strategy^46^,^47^,^48^. Therefore, while improved methodologies for manipulating rat zygotes and ES cells are facilitating the production of transgenic rats, the associated challenges have been immense, leading many to overlook rats as a potential model system for biomedical and translational science, and instead, relying on transgenic mice as the primary source of animal research model.

As methods for generating transgenic rats improve over time, the last decade has also witnessed a rapid advancement in genome engineering technologies. Among them, tools based on transposable element systems such as *piggyBac* and Sleeping Beauty, and engineered endonucleases such as ZFNs, TALENs and CRISPRs bursted onto the scene allowing manipulating and editing the genome with efficiency and precision never before possible. Because producing transgenic mice have traditionally been less difficult than rats, a large body of literature has accumulated demonstrating the ease with which the genomes of mice can be manipulated using these tools to, for example, reprogram somatic cells in into iPS cells, induce random mutagenesis, conduct precise genome editing through oligo replacement, and knock out gene function via indels^33^,^37^,^39^,^49^,^50^. Although much smaller in number relative to mouse models, transgenic rats have been generated using these tools as well^42^,^51^,^52^,^53^,^54^,^55^.

In spite of the great potential engendered in these newly emerging genome engineering tools, BAC transgenesis continues to be a method of choice for many. The advantages imbued in BAC-based transgenics stem from the large genomic insert size, encompassing one or more genes with essential regulatory elements for proper expression of the transgene, and insulating elements that curb epigenetic silencing due to position effect variegation^56^. Moreover, the time and cost effectiveness of modifying BACs have allowed for high throughput production projects such as the Gene Expression and Nervous System AtLas (GENSAT)^57^,^58^ and Knock Out Mouse Project (KOMP)^59^ which continue to play a pivotal role for enhancing our understanding of gene function and regulation. These are some of the examples delineating the importance and benefits attributed to generating model organisms using BAC transgenesis. Perhaps more importantly, for many research studies involving human therapy and disease modeling, BACs provide the most effective method of recapitulation^60^,^61^,^62^,^63^,^64^, as demonstrated by humanized *SIRPA* mice.

The major limitations of using BACs have been the cumbersome handling procedure and low insertion efficiency in the genome. A major breakthrough came in 2011 when Li and colleagues^65^ demonstrated for the first time that *piggyBac* TIR elements can be used to mobilize a fraction of BAC as large as 100 kb into the genome of mouse ES cells, and Suster and colleagues also showed in the same year BAC transposition in zebrafish using *Tol2* transposable elements^66^. In 2013, Rostovskaya *et al*. reported that BACs modified with *piggyBac* TIRs can be injected directly into mouse zygotes for efficient generation of founders carrying the transgene^67^. In rats however, the mediation by *piggyBac*, nor any other genome engineering tools, has yet to be reported for generating BAC transgenics.

Here, we aimed to examine whether *piggyBac*, CRISPR/Cas9 and TALEN mediated approaches may enhance *hSIRPA*-BAC transgenesis in the rat zygote. To do this, we strategically modified the BAC with elements compatible for recognition by these tools. Through this approach, we discovered that *piggyBac* provided an efficient approach for generating *hSIRPA*-BAC transgenic rats that are germline transmissible, and faithfully express functionally viable human SIRPα protein in the monocytes of F1 offspring. We have also demonstrated that monocytes from BAC transgenic rats express human SIRPα, as observed in human monocytes, indicating that regulation of expression by *hSIRPA*-BAC is conserved. Since rat monocytes from BAC transgenic rats express human SIRPα which interacts with human CD47, it is likely that the transgenic rat monocytes will receive phagocytosis inhibitory signals from human SIRPα. Immune humanization of immunodeficient rats has been reported to be undetectable^68^, and backcrossing with human *SIRPA* transgenic rats will likely increase efficiency.

By laying out a blueprint for generating *piggyBac* mediated *hSIRPA*-BAC transgenesis in rats, we provide an efficient tool in the hands of the research community, for expanding the existing platform for generating *hSIRPA*-BAC transgenic rats in various immune compromised strains^21^, as has been done for the mouse immune humanized models^13^,^20^. Humanized *SIRPA* transgenic rats will likely be useful in other biomedical and translational research arena as well, for example, a growing body of literature suggests the relevance of *SIRPA* in inflammation^69^, stem cells^70^, cancer^71^ and cell cycle regulation^72^.

Concomitantly with the submission of our manuscript, the genomic insertion of a BAC using the CRISPR/Cas9 approach has been described in the rat^41^. The authors describe the use of ssODNs with short homology sequences bridging BAC sequences (human SIRPA) and the genomic region into which the BAC was targeted (rat *Sirpa*). The efficiency of BAC transgenesis by this approach was comparable to that of the same BAC using classical transgenesis (13.3 and 9.2% of born animals, respectively). In contrast, the *piggyBac*-mediated approach increased the efficacy of BAC transgenesis compared to that of classical transgenesis (24 vs. 6.7%, respectively). BAC insertion using ssODNS and guide RNAs occurred in the targeted locus whereas *piggyBac*-mediated BAC transgenesis resulted in random insertions in TTAA sites. Nevertheless, the insertion site of the *piggyBac*-mediated BAC transgene can be easily determined by splinkerette PCR, and thus the risk of insertional mutations can be evaluated. It is surprising that Yoshimi *et al*.’s CRISPR/Cas9 approach using ssODNs resulted in BAC insertion into the targeted locus, whereas the use of longer homology arms in our study did not allow for targeted integration. The different outcome may be attributed to the differences in mechanisms underlying insertions using ssODNs versus homology arms. In the case of ssODNs, it is likely that the insertion involved ssODN-mediated nonhomologous end-joining^73^,^74^, whereas the homology arms may have required homologous recombination. These two repair pathways not only use completely different protein complexes but also occur at different times of the cell cycle (G0/G1 for NHEJ and G2/S for homologous recombination). Furthermore, it is conceivable that different loci (rat *Sirpa* vs. rat *Rosa26*) may be differentially susceptible to insertion of BACs by the two repair pathways. Importantly, researchers now have the choice of performing BAC transgenesis either using Cas9/ssODNs- or piggyBac-mediated integration, with each system having advantages and disadvantages. The CRISPR/Cas9 approach may be particularly attractive once optimal parameters for this approach have been established, as it conceivably allows for precise editing of the genome including the creation of models where the human gene replaces the mouse or rat homolog.

The report by Rostovskaya *et al*.^34^ was the first to demonstrate *piggyBac* mediated BAC transposition in mouse zygotes. Our manuscript shows that *piggyBac* transposition of BACs is also an efficient method in rats, resulting in increased transgenesis with typically a single copy insertion faithfully expressing the transgene. Moreover, the insertion sites are easily identifiable using simple PCR. Our results support the possibility that this approach may also be useful for generating transgenic animals in other mammalian species of biomedical or agricultural significance. We envision that in the near future, rapidly advancing technologies will allow for an efficient targeting of BACs into specific loci in the genome for the production of rat transgenics. Likewise, the emergence of newer, more advanced, technologies may no longer benefit from BAC-based transgenics for simple procedures such as knocking out genes or knocking in reporters. However, it is premature to disregard BACs as “obsolete” as recently claimed^75^. BACs have served an important function in science thus far, and we foresee continued relevance. Therefore, improving the method for generating BAC transgenic animals, such as the rat, using rapidly advancing genome engineering technologies can only benefit research, and lead us closer to translation and therapeutic applications.

## Material and Methods

### Conversion of *hSIRPA* BAC into a *piggyBac* transposon

Human BAC, RP11-887J4, carrying 176,233 bps of genomic DNA segment covering the SIRPA transcript coding sequence, as well as the region spanning 52,081 bps up- and 78,424 bps down-stream of the transcript start and end points, was selected for retrofitting with the *piggyBac* TIR elements. The RP11-887J4 BAC clone was acquired from BAC/PAC Resources (bacpac.chori.org), and retrofitted using the recombineering technology. The recombineering cassette carrying the TIR elements was generated by subcloning the 5′ TIR and 3′ TIR elements derived from pPB-CAG-EBNXN (Wellcome Trust Sanger Institute) into a pUC19 vector backbone with the spectinomycin resistance genes in between the TIR elements. The recombineered BAC clone was PCR and sequence verified.

### Modification of *hSIRPA* BAC with rat *Rosa26* homology arms flanking *Rosa26* or *AAVS1* sgRNA recognition sequences

The RP11-887J4 BAC clone was retrofitted with a recombineering cassette carrying 1049 and 1038 bp length rat Rosa26 homology arms flandking the Rosa26 sgRNA recognition sequence. The cassette was generated by PCR amplifying the 5′ and 3′ homology arms from CHORI230-418P10, for which one of the primers carried the Rosa26 sgRNA recognition sequence. The PCR amplified fragments were subcloned into a pUC19 vector backbone with neomycin resistance gene driven by PGK and EM7 as a selection cassette using the Gibson Assembly kit (NEB).

### HyPBase mRNA transcript preparation

To prepare the hyPBase mRNA transcript, pCMV-hyPBase (generous gift from the Wellcome Trust Sanger Institute) was digested with SalI restriction endonuclease, and cleaned up using the PCR Cleanup Kit (Macherey Nagel). 500 ng of the linearized plasmid was used as a template to transcribed *in vitro* using the mMESSAGE mMACHINE Kit (Ambion) according to the manufacturer’s instructions. The transcribed mRNA was purified using the MEGAclear Kit (Ambion).

### Zygote injections

The rats used in this work were from the Sprague-Dawley (SD/Crl) strain (Charles River, L’Arbresle, France). The study was approved by the Ethics Committee on Animal Experimentation of the Pays de la Loire Region, France, in accordance with the guidelines from the French National Research Council for the Care and Use of Laboratory Animals (Permit Numbers: APAFIS#692), as such, the methods themselves were conducted in “accordance” with the approved guidelines. The BAC constructs and hyperactive transposase were injected at different concentrations indicated in the results section (Tables 1 and 3). The BAC was diluted in a buffer containing 10 mM Tris-HCl, pH 7.5, 0, 1 mM EDTA, 30 uM spermine, 70 uM spermidine, 100 mM NaCl^76^. The BAC were handled gently and kept on ice during microinjection. Fertilized 1-cell stage embryo collection and sequential microinjection into the male pronucleous and into the cytoplasm using different BAC and hyPBase concentrations, Cas9 protein and sgRNA for rat Rosa26 (sgRNA and Cas9 protein incubated before microinjection), as well as TALENs mRNA for rat *Rosa26* and donor DNA as previously been described in detail^42^. We demonstrated that Cas9 protein was more efficient than its mRNA to generate knockin by homologous recombination^42^. Microinjected embryos were maintained in 5% CO_2_ at 37 °C for at least 1 h. Surviving embryos were then implanted on the same day of microinjection in the oviduct of pseudo-pregnant females (0.5 days post coitum) and allowed to develop until embryonic day 15 or until normal delivery. Offspring were obtained by crossing BAC positive founders with wild-type rats.

### Genotyping analysis

Embryos or pups were identified by PCR using the following primers: 5′-CTCTACGCGCTTTCTTGTCC-3′ and 5′- AACGTCAGCCTCCAGGTATG-3′. The standard amplification profile consisted of 5 mn at 95 °C, 2 mn at 62 °C followed by 35 cycles of 30 s at 72 °C, 10 s at 95 °C, 10 s at 60 °C and 3 min at 72 °C. Amplicons were analyzed using microfluidic capillary electrophoresis^77^.

### Splinkerette PCR for sequencing of transposon integration sites

Genomic DNA was isolated and digested with Sau3AI. The digested genomic DNA was ligated to 50 nM of splinkerette adaptors using T4 ligase and ligation buffer from NEB. Nested PCR was used to amplify the genomic DNA sequence adjacent to the 5′ and 3′ TIR ends. The PCR products were sequenced to determine the precise transposition sites.

### Realtime Quantitative PCR (qPCR)

Using 20 ng of isolated genomic DNA, qRT-PCR reactions were performed with SYBR Green PCR Master Mix (Applied Biosystems) according to manufacturer’s instructions. 200 nM of primer pair used for genotyping the presence of the human SIRPA gene was used for qRT-PCR. The Ct values were calculated using Applied Biosystems’ SDS2.4 software. To determine the copy number of the transpositioned BACs, the Ct values derived from the primer pair used for amplifying the human SIRPA gene was normalized to the GAPDH gene in the rat genome.

### Flow cytometry

Rat peripheral blood leukocytes were isolated from heparinized blood by red blood cell lysis. hSIRPA was detected using an anti-human SIRPA monoclonal antibodies (clones REA144, Miltenyi or SE5A5, BD Biosciences, both IgG1). Recombinant human CD47-Fc (hCD47-Fc, R&D Systems) was also used to detect human SIRPA expression since it does not bind to rat SIRPA. Rat SIRPA was detected using anti-rat SIRPA-FITC monoclonal antibody (OX41, European Collection of Cell Culture). Several isotype negative controls were used: APC-mouse IgG1, FITC-mouse IgG2a, PE-mouse IgG1 (BD Pharmingen). All monoclonal antibodies, hCD47-Fc and human IgGs were used at 10 ug/ml. Fluorescence was analyzed with a FACSVerse flow cytometer (BD Biosciences), and FlowJo software was used to analyze data.

## Additional Information

**How to cite this article**: Jung, C. J. *et al*. Comparative Analysis of *piggyBac*, CRISPR/Cas9 and TALEN Mediated BAC Transgenesis in the Zygote for the Generation of Humanized *SIRPA* Rats. *Sci. Rep.* **6**, 31455; doi: 10.1038/srep31455 (2016).

## Supplementary Material

Supplementary Information

## Figures and Tables

**Table 1:** Transposition of BACs in Rat Zygotes Mediated via Hyperactive *piggyBac* Transposase.

BAC DNA[ng/ul]	hyPBase[ng/ul]	Number of Embryos Injected	Number of Embryos Transferred	Number of Pups	% Number of Pups Relative to Number of Embryos Transferred	Number of Pups PCR Positive	% PCR Positive Pups Relative to Embryos Transferred	% PCR Positive Pups Relative to Total Number of Pups
3	100	41	19	7	36.8%	1	5.3%	14.3%
3	50	197	99	13	13.1%	1	1.0%	7.7%
2	25	276	181	25	13.8%	6	3.3%	24.0%
	**Cas9 [ng/ul]**							
2	25	124	78	15*	32.1%	1*	1.3%*	6.7%

^*^Refers to pups at day 15.

**Table 2:** Analysis of germline transmission efficiency in founders derived from *piggyBac* mediated hSIRPa BAC transposition.

hSIRPa-BAC-TIRs Founders	Number of pups	Number of PCR positive pups
1.6	20	13
4.2 (fragmented BAC)	25	5
5.4	22	0
5.5 (random BAC insertion)	22	0
5.7	27	2
8.1‡	NA	NA
9.2	7	0
9.4	20	8

^‡^Refers to founders that died before mating.

**Table 3:** Targeting hSIRPa-BAC into the Rosa26 locus using CRISPR/Cas9 and TALENs.

BAC DNA	BAC [ng/ul]	sgRNA + Cas9 protein	TALENs	Number of Embryos Injected (%viable)	Number of Embros Transferred	Number of E15 Embryos (% Relative to Transferred)	Number of Embryos PCR Positive (% Relative to Total E15)	% NHEJ Positive (% Relative to Total E15)
RP11-887J4 Rat-Rosa26^−^HAs with sgRNA Target Sequence	2	3 uM + 3 uM	NA	194 (76.3)	148	60 (40.5)	1 (1.7)	22 (36.7)
RP11-887J4 Rat-Rosa26^−^HAs without sgRNA Target Sequence	2	3 uM + 3 uM	NA	125 (69.6)	87	28 (32.2)	1 (3.6)	12 (42.8)
RP11-887J4 Rat-Rosa26^−^HAs with sgRNA Target Sequence	2	NA	5 uM + 5 uM	X177 (68.9)	122	39 (32.0)	0 (0)	9 (23.1)
RP11-887J4 Rat-Rosa26^−^HAs without sgRNA Target Sequence	2	NA	5 uM + 5 uM	100 (66.0)	66	13 (19.7)	0 (0)	4 (30.8)
