# Supplementary material for: Comparative Analysis of piggyBac, CRISPR/Cas9 and TALEN Mediated BAC Transgenesis in the Zygote for the Generation of Humanized SIRPA Rats
Source: Sci Rep. 2016 Aug 17;6:31455. doi: 10.1038/srep31455 (PMC4987655; doi:10.1038/srep31455)
Supplement: Supplementary Information [file srep31455-s1.pdf]

Comparative Analysis of piggyBac, CRISPR/Cas9 and TALEN Mediated BAC Transgenesis in the Zygote for the Generation of Humanized SIRPA Rats

Chris J. Jung, Séverine Ménoret, Lucas Brusselle, Laurent Tesson, Claire Usal, Vanessa Chenouard,Séverine Remy,Laure-Hélène Ouisse, Nicolas Poirier, Bernard Vanhove, Pieter J. de Jong, Ignacio Anegon

Supplementary Figure 1

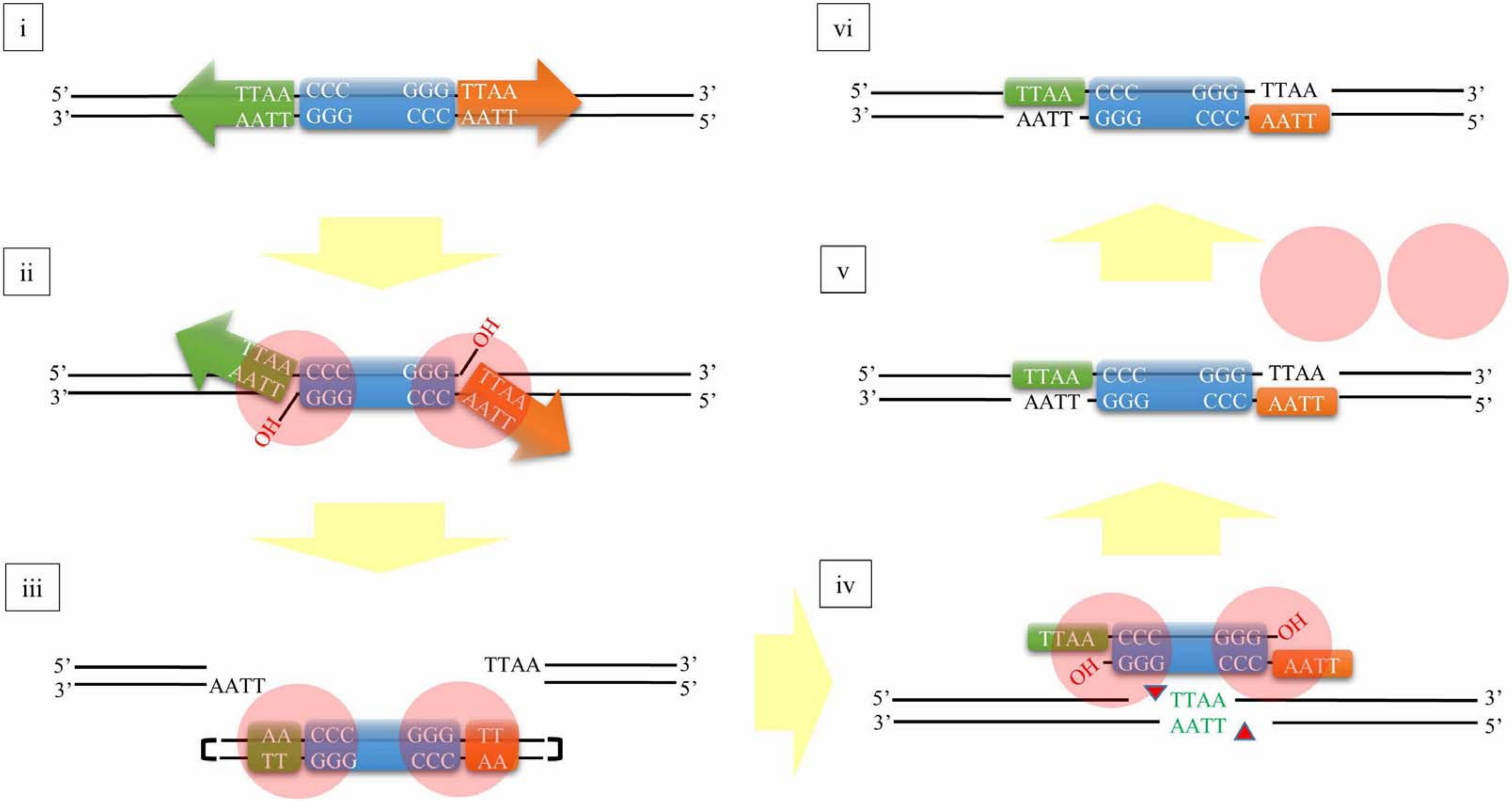

Supplementary Figure 1. The mechanism of *piggyBac* transposition. (i, ii) The transcribed transposase proteins bind to the inverted repeats in order to induce nicks at both ends of the transposon, exposing the 3’ hydroxyl group and TTAA tetranucleotide overhang in the opposite strands. The 3’ hydroxyl end launches a hydrophilic attack on the TTAA overhang, creating a hairpin formation, which initiates the release of the mobile DNA from the genome. (iii) Once released, the hairpin structure is resolved by leaving TTAA overhang at the 5’ ends of the transposon, and 3’ hydroxyl group exposed at the opposite strands. (iv) During this process, the transposase proteins identify other TTAA sequences in the genome, and likewise, induce nicks and hydrophilic 3’ hydroxyl attacks on the TTAA tetranucleotide sequences. The resolution of the hairpin formation in the genome involves target joining of the 3’ hydroxyl groups at the transposon ends, with the 5’ staggered TTAA overhanging sequence in the genomic DNA. Through this process, the mobile DNA gets inserted into the genome, by duplicating the TTAA sequence, and positioning itself such that each tetranucleotide sequence ends up residing at opposite ends.

Supplementary Figure 2

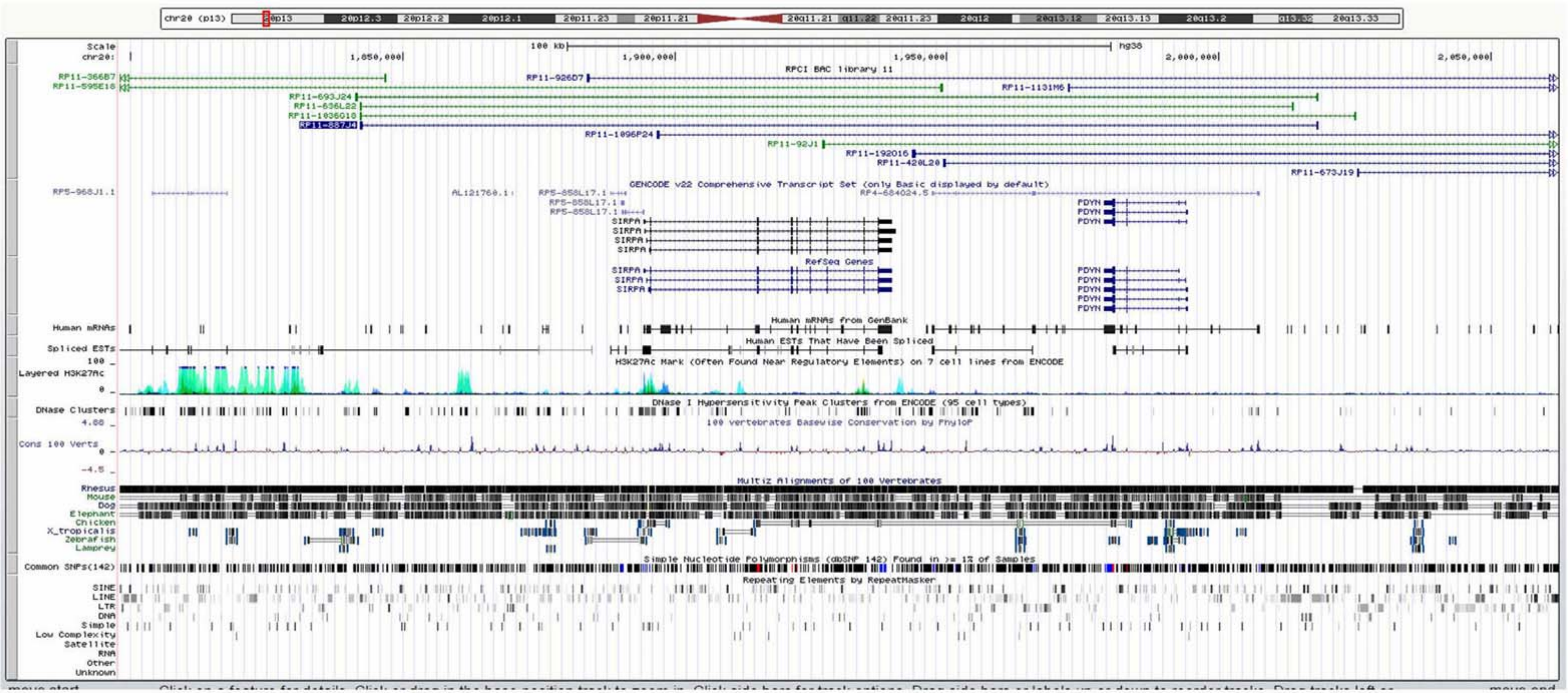

Supplementary Figure 2. Description of RP11-887J4. Human *SIRPA* carrying BAC clones were identified using the UCSC Genome Browser (https://genome.ucsc.edu/). Among the BAC clones available, RP11-887J4 was selected because it contains a large segment of insert DNA up and downstream of the *SIRPA* gene. The position of genomic insert carried in RP11-887J4 clone is chr20:1842086-2018318, and the size is 176233.

Supplementary Figure 3

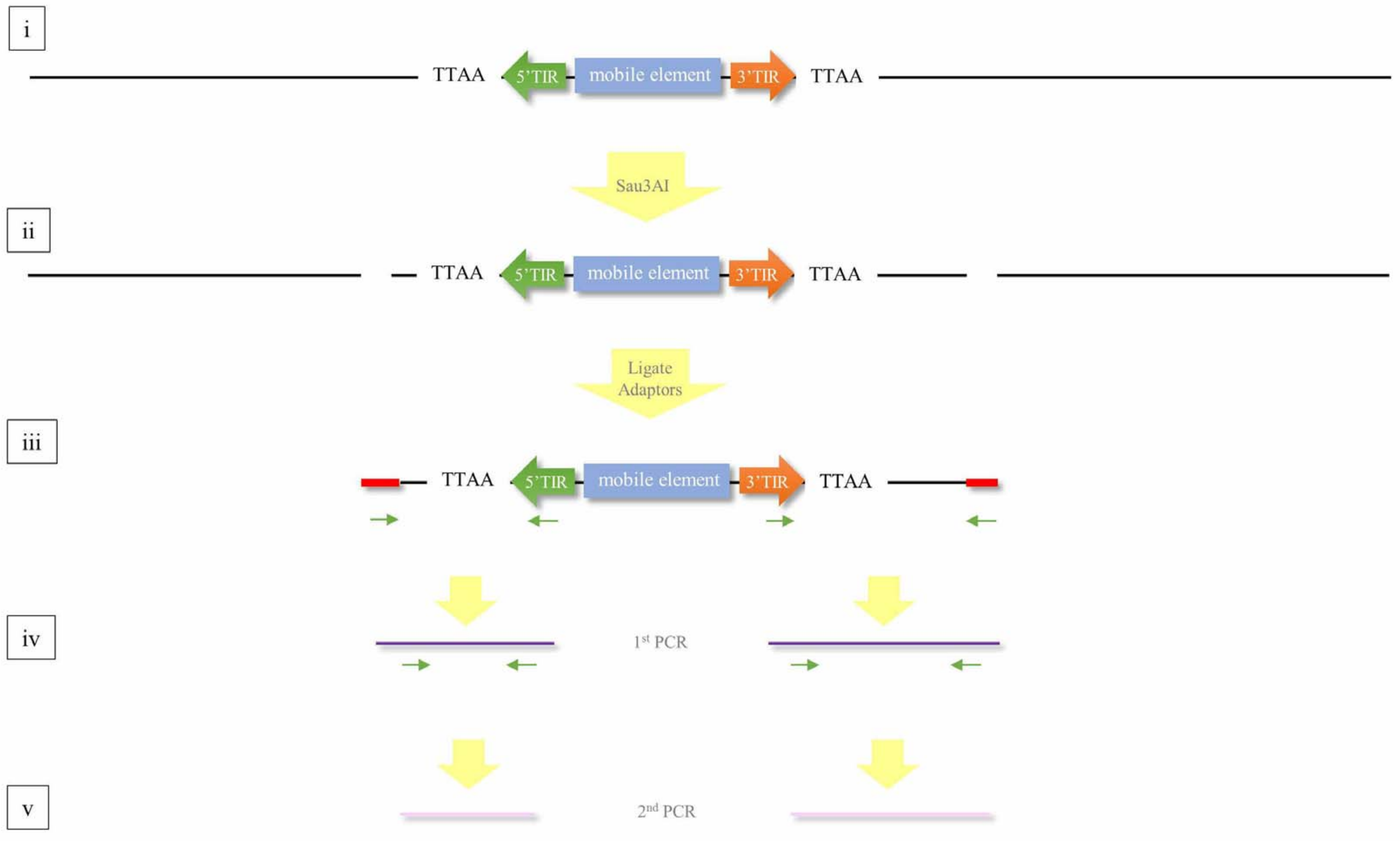

Supplementary Figure 3. Splinkerette PCR. (i, ii) Genomic DNA is digested with Sau3AI. (iii, iv) Adaptors are ligated to the ends of the digested DNA. Nested PCR using primers binding to the adaptors and TIRs. (v) PCR products are sequenced to determine the insertion site.

Supplementary Figure 4

A

B

C

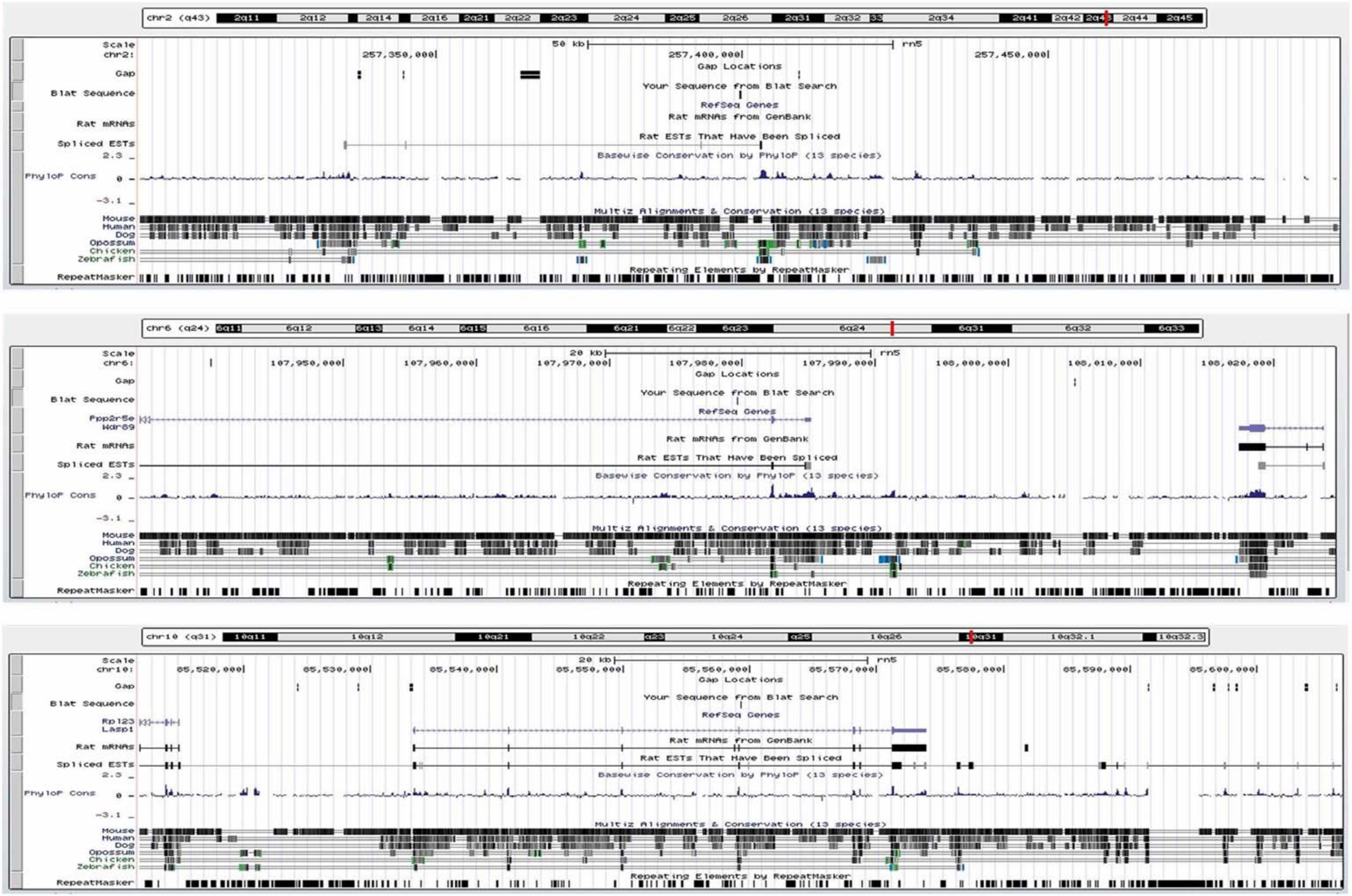

Supplementary Figure 4. ***piggyBac* transpositioned BACs.** (A) Insertion sites for 1.6 (B) 5.4 (C) 5.7.

Supplementary Figure 4

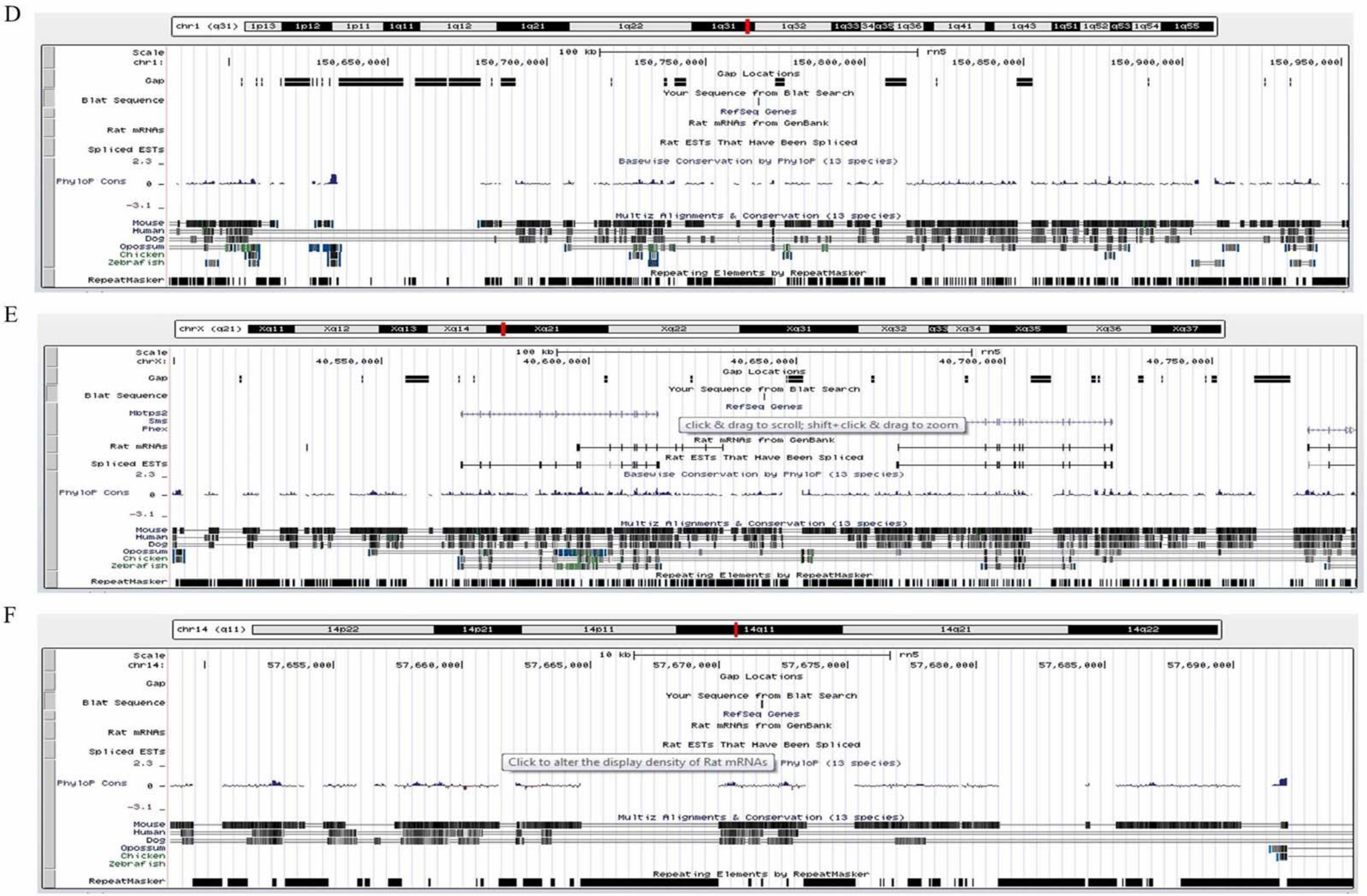

Supplementary Figure 4. ***piggyBac* transpositioned BACs.** (D) Insertion sites for 8.1 (E) 9.2 (F) 9.4.

Supplementary Table 1. Primer Sequences

Primer Name	Sequence 5' --> 3'	Product Size
hSIRPa -F	CTCTACGCGCTTTCTTGTCC	201
hSIRPa -R	AACGTCAGCCTCCAGGTATG	
rGAPDH-F	TGTGAGGGAGATGCTCAGTG	204
rGAPDH-R	CCGTTGTCCCAATCTGTTCT	
hSIRPa (RP11-887J4)-F1	GACCCAGCAATTCCACATCT	237
hSIRPa (RP11-887J4)-R1	ATCCATGCTGTACCGTGTGA	
hSIRPa (RP11-887J4)-F2	CTGACTCTCTGCCCATCACA	233
hSIRPa (RP11-887J4)-R2	GGGCTCCCCTCTTCATACTC	
hSIRPa (RP11-887J4)-F3	TGATCTTGGCTCACTGCAAC	197
hSIRPa (RP11-887J4)-R3	AAGTGGGTGGATCACCTGAG	
hSIRPa (RP11-887J4)-F4	TAAGAGCCGTGGAAAGTGCT	231
hSIRPa (RP11-887J4)-R4	CAGGCTTCTGGATTCTCAGG	
hSIRPa (RP11-887J4)-F5	ACTGTTGGCCCCAGTAACAG	247
hSIRPa (RP11-887J4)-R5	CACTGGGCTCAGGAGTTAGC	
hSIRPa (RP11-887J4)-F6	ATCCGGTTAATGTCCCACAA	230
hSIRPa (RP11-887J4)-R6	AGGCAGTGCTTCATCTCGTT	
hSIRPa (RP11-887J4)-F7	CAGCCCCTATTCAAGATGGA	245
hSIRPa (RP11-887J4)-R7	GGAGGTGCTATGCGTGAAAT	
hSIRPa (RP11-887J4)-F8	CTCAAAGGAAAGCCGACAAG	236
hSIRPa (RP11-887J4)-R8	GGCAACAGGGAGTGTCATTT	
hSIRPa (RP11-887J4)-F9	AGAGACGGCCTCTGTTTCAA	227
hSIRPa (RP11-887J4)-R9	TCCTTTCCTTCCCAGTTGTG	
hSIRPa (RP11-887J4)-F10	AGAGCGAGACTCCGTCTCAA	187
hSIRPa (RP11-887J4)-R10	GAGGTGTTCAGGGGCAATAA	
